# The Harveian Oration

**Published:** 1898-10-22

**Authors:** 


### THE HARVEIAN ORATION.

The Harveian Oration, which was delivered by Sir Dyce Duckworth before the Royal College of Physicians on Tuesday afternoon, was a model of what such a discourse should be. Its words were well weighed, its sentences were carefully rounded, and yet the fire of a righteous indignation and the earnestness of a strong conviction were not lacking when the orator came to speak of matters of current interest, and to denounce the manner in which a great sanitary question has lately been treated by the Government. Sir Dyce Duckworth devoted himself mainly to three points; the praise of good men, men who, by their devotion to the College and its interests, by their good example, and by their benefactions, had done so much not only to raise and maintain the position of the Royal College, but to elevate the tone of medical practice throughout the country; the insistence upon that upon which Harvey himself had laid such stress, namely, the importance of seeking truth by actual experiment; and, lastly, the duty which lay upon them of maintaining among themselves a high standard of duty and of honour. Sir Dyce Duckworth laid especial stress upon the fact that it was by their virtues and their character as well as by their scientific attainments, by the lives they had led, as well as by the work they had done, that they had helped to establish and make firm the influential position of their college. Touching upon the search for truth by aid of experiment, he maintained that the results of laboratory investigations, when full and mature, must always tally with those obtained by clinical experience, and he threw mighty scorn upon those who, on the strength of more or less isolated experiments in the laboratory, presumed to set themselves in opposition to conclusions reached by patient observation of actual disease made by physicians on whose intellect, honesty, and acumen no man could throw any doubt. After tearing to tatters the objections which, on the strength of certain "experiments" conducted in America, had recently been raised to the use of alcohol in acute disease, Sir Dyce Duckworth turned to an examination of some of the more recent developments of bacteriological science, and laid great stress upon the preponderating influence which was exercised by what must be called the personal factor in the production of disease. True as it was that the microbe and its toxins were essential to the production of certain maladies, it was also true that there existed in different individuals varying constitutional conditions or diatheses predisposing to, or preventing, the injurious action of the micro-organisms in question. The observations of the older physicians as to struma—its characteristics and its tendencies—were as true to-day as ever they were; and while we must accept the tubercle bacillus as an essential factor in tuberculosis, no true conception of the disease as a whole could be obtained without recognising the influence upon its progress of special constitutional conditions, one of which was that of struma. And what was true of tuberculosis was true also of other diseases. He then went on to speak of the important observations of Dr. Monckton Copeman in regard to vaccination and the production of glycerinated lymph, and gave solemn warning of the danger to which the country was being exposed as the result of recent legislation. Finally, the orator, in well-chosen words, urged that the college should always maintain its high standard of dignity and honour, and in his closing sentences he offered graceful homage to the great man whose memory they that day kept alive. As we listened to this address, we could not but feel that much that is best in the Royal College of Physicians was well represented by Sir Dyce Duckworth. In dignity of words and gesture, in his selection of what was most noble for the greatest praise, in his way of measuring the most recent scientific investigations by the light of an older experience, and by his constant insistence on the necessity of high character if the physician were to exercise his proper influence upon his generation, the Harveian Oration of this year will take a high place among such compositions.

